# New Method of Arresting Post Partum Hemorrhage

**Published:** 1880-05-20

**Authors:** 


### *NEW METHOD OF ARRESTING POST PARTUM HEMORRHAGE.*

---

We find in The Practitioner a Paper read before the New York Academy of Medicine, by Isaac E. Taylor, M.D., of New York.

1st.—*Flagellation of the Child's Back previous to its complete Delivery, as a Preventive of Uterine Hemorrhage.*

2d.—*Flagellation of the Abdomen of the Woman after the Delivery of the Placenta, as a Substitute for the Introduction of the Hand into the Uterine Cavity.*

The writer thus states his two propositions:

*First.*—Flagellation or spanking the child's back moderately, every now and then, after the delivery of the shoulders, permitting the breech and the extremities of the child to remain in the vagina, and the feet thus placed in apposition with or in the cervix uteri, remaining for fifteen or twenty minutes or more without being withdrawn. Pressure over the uterus by the hand is to be avoided till the delivery of the child, which should be slow and gradual, as it might effect the delivery of the child before we have gained our object, and at the same time the spanking should be quick but gentle, and not too harsh, and continued until the delivery of the child is completed.

*Second.*—After the delivery of the placenta, should hemorrhage occur, expose the abdomen, and flagellate it with a towel doubled up, the ends held in the hand, saturated or not with ice water. Several rapid and powerful strokes should be made, when the unrecognized uterus will be almost immediately felt contracting or contracted, no matter how profuse or rapid the flow may be. In one instance, having ocular demonstration after the delivery of the placenta, the stream of blood was as large, full and rapid as that which flows from a Croton faucet.

Should uterine contraction ensue and relaxation take place, a milder application of the same means may be resorted to, till the contraction is deemed secure, and other measures adopted if necessary.

There can be no procrastination or temporizing action in these sudden and violent cases. The appearance of the method to those present, or to the patient herself, if conscious, with the suddenness and rapidity of its application, may seem harsh, abrupt and unnecessary. We have, however, nothing to do with appearances or feelings in such critical emergencies. We are imperatively reminded that life or death is swaying in the balance. Duty commands decided and prompt action. By this procedure I have in some instances had the gratification of feeling the apparently lifeless organ fold itself up under the touch, the uterus contracting or contracted, and our patient's life safe certainly for the time being. Under such circumstances, hot or cold water injections, as well as the hand internally, has, in many instances, failed to arouse into contraction the perfectly atonic or moribund organ.

After contraction has once been secured, then that treatment which the views or experience of the medical attendant may elect can be pursued, whether by hot water or cold, externally or internally, or mixed with other substances, or by tincture iodine or sulphate of iron, accomnied with the ordinary and usual manipulations externally over the uterus.

The principles upon which this method is based may be gathered from the following quotation :

"After the delivery of the child, it is a recognized fact that the tender surfaces of the vagina and cervix uteri become more instantly excitor, and when they are touched the motor irritation of the uterus will be more susceptible and quickly provoked and more rapid than when acting alone. The vaginal and the cervical excitor nerves are not only in relation to the true spinal marrow, or medulla spinalis, but they are in consonance with the medulla oblongata and cerebellum. The excitation of the vaginal and cervical nerves, therefore, through reflex action, arouses the motor nerves going to the body of the uterus, and they become instantaneously engaged, or brought into action, by the irritation or titillation of the child's feet when the back is slapped, aided through the distension of the vagina by the breech of the child.

"The physiological views presented and so generally accepted respecting the innervation of the uterus, have lately been investigated and attested by Hauk and Halle, by numerous experiments on animals, affirming that the nerve centres regulating the uterine contractions are centered in the cerebellum, medulla oblongata, and the lumbar portion of the spinal cord. We recognize, therefore, that superficial excitation electrically of the cerebral convolutions causes no uterine contraction.

" Excitation of the vagus, when alone, has no effect on the uterus.

" Excitation of the cord, when the sympathetic and the sacral nerves are divided, has no effect on the uterus.

" Excitation of the uterus itself calls forth only local muscular movement, and no strong general contraction of the organ. Unless, therefore, the reflex nervous system can be brought into action, when the general nervous system has suffered or become prostrated through a tedious and prolonged labor, or idiosyncrasy of constitution or exhaustion consequent on profuse flooding, giving rise, as it will in some cases, to almost complete anæmia of that organ—if, under these circumstances, we cannot rouse into action that vitally important system, and produce contraction of the uterus quickly and promptly, but rest content with the direct method, or irritation through the uterine nerves almost solely, whether by external or internal means, an unfavorable issue dependent upon the want of excitation, whatever that may be, of the reflex system will occur, and the patient will be more likely to die. I do not forget that another external and important physical agent is resorted to sometimes, originally suggested by Gooch—that is cold water dashed or poured from a height, on the abdomen when exposed—one to all appearance closely allied in principle to the method I propose. The shock, however, produced by these means may be so transient as to create no permanent effect in some cases."

The following cases are given as illustrative of this plan.

*Case* 1.—A few years since I was engaged to act in consultation with Dr. G—— in the case of Mrs. V——. Her confinements had been of the ordinary character, accompanied by severe pains, though the labor was not prolonged. This was her fourth confinement. Profuse flooding occurred always after the birth of the child, and the patient was left in a critical state for some time afterward. There was also considerable difficulty in arresting them. The child was above the average weight. I was notified of the event a few hours after the commencement of the labor. When I arrived I was ushered into a room adjoining hers. I was informed shortly after that the labor was progressing favorably—natural presentation and so on. An hour afterward, judging from the strenuous efforts the patient was making and the nature of her pains, the delivery must be near at hand. It was evident if my services were to be of any value, with the view I held, I could be of no assistance in her case unless Nature was more provident this time than on previous occasions. I therefore went into the chamber, and found the head of the infant was born and the shoulders about to be delivered. At this stage I requested, after the shoulders had passed, that the cord should be drawn down, and the breech and extremities of the child detained in the vagina and cervix, the uterus not to be pressed upon nor held. The back of the child was then spanked sharply and quickly for twenty minutes or more, at intervals. There was some slight evidence of hemorrhage, and no more. The child was then slowly delivered; the uterus grasped and well contracted, though not hard. Occasional relaxation and contraction, but no flooding. In ten minutes after the placenta came away safely. Remained for one hour, and everything was natural and safe. Morning visit all well.

*Case* 2.—I was requested to attend Mrs. L—— with her fifth confinement, receiving the information how critical her confinements had been, consequent not only on the tedious labor but the excessive flow which followed the delivery of the placenta. The labor was natural, though as formerly tedious for several hours. Chloroform was requested not to be given, and to that I most cheerfully assented. The head of the child was safely delivered by enucleation, the shoulders came gradually and slowly through the vulva. The cord drawn down; breech and extremities remaining; uterus not held; child's back flagellated occasionally, and not withdrawn for nearly half an hour; every time the child was whipped, the child would gasp. The delay of the delivery of the child created anxiety on the part of the mother, and she asked why the baby was not removed. The sensation of the child's feet she did not like pressing against the womb. The placenta escaped very soon after the delivery. No flooding ensued but what is incident to delivery in ordinary cases. The getting up was excellent.

*Case* 3.—I was requested many years ago to hasten immediately to meet Dr. Robeson in the case of Mrs. E——, who resided but a short distance from my house, July 4, 1846. She had just been delivered, and the placenta followed soon after. She was blanched, rolling from side to side; gasping, sighing and twitching of the muscles of the face; abdomen as large almost as at full term. The uterus was instantly grasped with the left hand, the right loosing the clots and removing them from the vagina and uterus. Immediately the uterus was flagellated smartly several times, when contraction ensued. This course was pursued moderately and occasionally for several minutes, while the ordinary means were also attended to. No relaxation took place, although she was watched closely for two hours or more. Brandy injections for rectum were given and nourishment. Nothing unfavorable occurred, but a slow convalescence.

*Case* 4.—Mrs. V——. In consultation with Dr. S——, in a case of kyphotic pelvis, having an outlet in the inferior strait of a transverse diameter of 1⅞ in. between the tuber ischia and antero posterior 2½ inches. After the delivery of the child by cephalotropy, while awaiting the delivery of the placenta, a few moments after the expulsion a rapid and full stream of blood issued from the vagina, as though a water faucet had been open or turned on. Instantly I arose from my seat and gave the bared abdomen several rapid and strong flagellating strokes over the uterine region with a towel doubled up, wet with ice water, and as instantaneously was the flooding arrested. The round, globular uterus was then felt contracting under the hand. An electric shock could not have acted with more or as much effect so successfully, to the surprise of all present.—*Independent Practitioner.*

